# DDI-PULearn: a positive-unlabeled learning method for large-scale prediction of drug-drug interactions

**DOI:** 10.1186/s12859-019-3214-6

**Published:** 2019-12-24

**Authors:** Yi Zheng, Hui Peng, Xiaocai Zhang, Zhixun Zhao, Xiaoying Gao, Jinyan Li

**Affiliations:** 1 Advanced Analytics Institute, Faculty of Engineering and Information Technology, University of Technology Sydney, 15 Broadway Ultimo, Sydney, 2007 Australia; 2 School of Engineering and Computer Science, Victoria University of Wellington, Cotton Building, Kelburn Campus, Wellington, 6140 New Zealand

**Keywords:** Drug interaction prediction, Positive-unlabeled learning, Reliable negative samples

## Abstract

**Background:**

Drug-drug interactions (DDIs) are a major concern in patients’ medication. It’s unfeasible to identify all potential DDIs using experimental methods which are time-consuming and expensive. Computational methods provide an effective strategy, however, facing challenges due to the lack of experimentally verified negative samples.

**Results:**

To address this problem, we propose a novel positive-unlabeled learning method named DDI-PULearn for large-scale drug-drug-interaction predictions. DDI-PULearn first generates seeds of reliable negatives via OCSVM (one-class support vector machine) under a high-recall constraint and via the cosine-similarity based KNN (k-nearest neighbors) as well. Then trained with all the labeled positives (i.e., the validated DDIs) and the generated seed negatives, DDI-PULearn employs an iterative SVM to identify a set of entire reliable negatives from the unlabeled samples (i.e., the unobserved DDIs). Following that, DDI-PULearn represents all the labeled positives and the identified negatives as vectors of abundant drug properties by a similarity-based method. Finally, DDI-PULearn transforms these vectors into a lower-dimensional space via PCA (principal component analysis) and utilizes the compressed vectors as input for binary classifications. The performance of DDI-PULearn is evaluated on simulative prediction for 149,878 possible interactions between 548 drugs, comparing with two baseline methods and five state-of-the-art methods. Related experiment results show that the proposed method for the representation of DDIs characterizes them accurately. DDI-PULearn achieves superior performance owing to the identified reliable negatives, outperforming all other methods significantly. In addition, the predicted novel DDIs suggest that DDI-PULearn is capable to identify novel DDIs.

**Conclusions:**

The results demonstrate that positive-unlabeled learning paves a new way to tackle the problem caused by the lack of experimentally verified negatives in the computational prediction of DDIs.

## Background

Drug-drug interactions refer to the efficacy change of one drug caused by a co-administration of another drug. DDIs may occur when two or more drugs are taken together or concomitantly. DDIs account for around one-third of all adverse drug reactions [1–3], leading to significant morbidity and mortality worldwide [4]. Currently a few DDIs are identified via wet-lab experiments, however, a large number of DDIs remain unknown [5]. Thus, there is an urgent need to detect potential DDIs to reduce patients’ risks and economic costs.

Conducting experimental trials to detect potential interactions between a great number of drug pairs is unrealistic due to the huge time and monetary cost. Recently, several computational methods have been successfully applied to detect DDIs. Here, we categorize these methods roughly into three categories: similarity-based methods, knowledge-based methods, and classification-based methods.

The similarity-based methods assume that drugs with similar properties tend to interact with the same drug [6]. Based on this assumption, different drug similarity measures have been designed employing various drug properties. Vilar et al. measured the drug similarity as the Tanimoto coefficient between molecular fingerprints [6] and between interaction profile fingerprints of drug pairs [4]. Gottlieb et al. [7] built their DDI predictive model by integrating seven drug similarity measures, namely chemical structure similarity, ligand similarity, side-effect similarity, annotation similarity, sequence similarity, closeness similarity in the protein-protein network, and Gene Ontology similarity. By using the drug-drug similarity indirectly, Zhang et al. [8] designed a label propagation framework to predict DDIs based on drug chemical structures, labeled side-effects, and off-labeled side-effects. Similarity-based methods have achieved remarkable prediction performance, however, interactions for drugs lacking similarity information cannot be predicted. In addition, the assumption of similarity-based methods has one limit: dissimilar drugs may interact with the same drug.

The knowledge-based methods detect DDIs from scientific literature [9], electronic medical records[10], and the Food and Drug Administration Adverse Event Reporting System (FAERS) [11, 12]. He et al. [9] presented a Stacked generalization-based approach for automatic DDI extraction from biomedical literature. Tatonetti et al. [11] identified drug interactions and effects from FAERS using statistical methods. They found that interaction between paroxetine and pravastatin increased blood glucose levels. Knowledge-based methods rely on the accumulation of post-marketing clinical evidence. Consequently, they are incapable to detect all DDIs and cannot warn the public of the potentially dangerous DDIs before drugs reach the market.

Classification-based methods formulate DDI prediction as a binary classification task. Cami et al. [13] represented drug-drug pairs as feature vectors using three types of covariates from their constructed pharmacointeraction network. Then they defined the presence or absence of interactions as labels and finally built logistic regression models for predictions. Cheng et al. [5] encoded each drug pair as a 4-dimensional vector of four different similarities, and employed five classical prediction algorithms for predictions. Compared with similarity-based methods and knowledge-based methods, classification-based methods don’t have the assumption limitation or dependence on evidence accumulation. Nevertheless, two classes of data are required for classification methods: positive samples and negative samples. Existing classification-based methods used drug-pairs known to interact as positive samples, and other unlabeled drug-pairs as negative samples [5, 13]. These unlabeled drug pairs may include a considerable number of real positive samples which can degrade the prediction performance.

From the above survey, it is understood that similarity-based methods and knowledge-based methods are limited to their application ranges, while classification-based methods are lack of reliable negative samples. In this work, we explore an advanced learning technique named positive-unlabeled learning (PU learning) to solve the problem of lacking negative samples for the classification-based methods.

### PU learning and our new ideas

PU learning is to learn from the positive samples and unlabeled samples. PU learning has been successfully applied in several bioinformatic research fields, such as disease-gene association identification [14, 15], drug target detection [16] and glycosylation site prediction [17], and achieved remarkable performances. However, this advanced learning technique has not been explored enough in the prediction of drug interactions.

Conventional PU learning algorithms usually consist of two steps: the first step is to identify reliable negative samples from the unlabeled samples; the second step is to construct classifiers based on positive samples and identified reliable negative samples for subsequent predictions. The difference among different PU learning algorithms lies in different strategies used in the first or second step. In the first step, the spy strategy [18], 1-DNF [19], Rocchio [20] and Naive Bayesian (NB) [21] are widely used. The spy strategy selects a certain number of positive samples randomly as spies and puts them into the unlabeled samples first; then it determines the threshold of reliable negative samples (RNSs) under the condition that most spies are truly predicted as positives. The 1-DNF strategy extracts the features of positive samples and then selects RNSs which don’t have the positive features. Rocchio and NB first label validated positive samples as +1 and unlabeled samples -1 to train the Rocchio and NB classifier respectively. Then the trained classifier is employed to classify unlabeled samples. Those unlabeled samples which are classified as negatives are taken as RNSs. In the second step, Expectation Maximization (EM) and Support Vector Machine (SVM) are commonly used. Most conventional PU learning algorithms are designed for text classification, thus there are barriers to apply them directly to DDI predictions.

Apart from the above methods, clustering provides another solution to identify likely negatives from the unlabeled data. For example, Hameed et al. [22] successfully improved the clustering approach Self Organizing Map (SOM) for drug interaction predictions. However, they only obtained 589 inferred negatives after clustering, which is much less than the validated 6,036 positives (i.e., validated DDIs), let alone all potential negatives ($C_{548}^{2} - 6,036 = 143,842$) of their 548 drugs. Performing cross-validation directly on the very few negatives are incapable to convince readers of the generalization of their methods. Inspired by the clustering process of *k*-means a typical clustering method, we find a possibility to infer reliable negative samples via ranking of KNN. If we treat “positives" and “negatives” as two clusters, *k*-means clusters samples into “positives" if they are close to positives. Samples far from positives will be clustered as negatives. Therefore, we can use KNN to measure the distances between unlabeled samples and labeled positives. Unlabeled samples far from positives are inferred negatives.

One-class Support Vector Machine (OCSVM) [23] has been widely used for classification in the absence of positive or negative samples [24]. It learns a hypersphere to describe the training data and ensures most training data are in the hypersphere. OCSVM requires one-class data only, thus it is an ideal technique to identify reliable negatives in the PU learning context.

In this work, we design a novel two-step PU learning approach for drug-drug interaction predictions (DDI-PULearn hereafter). In the first step, DDI-PULearn infers highly-reliable negative sample (RNS) seeds using two techniques OCSVM and KNN. To be specific, DDI-PULearn learns an OCSVM hypersphere from all labeled positive samples (i.e., validated DDIs) with a high-recall (>0.95). Then DDI-PULearn predicts labels for all unlabeled samples and adds the predicted negatives to the RNS seeds. Meanwhile, DDI-PULearn infers several reliable negative samples using the KNN strategy and adds them to the RNS seeds. In the second step, DDI-PULearn identifies all reliable negatives from the remaining unlabeled samples using SVM trained by the RNS seeds and labeled positives iteratively. The labeled positives and identified RNSs are finally used for prediction and validation. The performance of DDI-PULearn is evaluated on simulated DDI prediction for 548 drugs. Comparison experiments with the two baseline methods and five state-of-the-art methods both demonstrate the superior performance of DDI-PULearn.

## Results

We first report the number of components for PCA. Then we present the prediction performances under different representations of DDIs using multi-source drug property data. Following that, we show the performance improvement brought by reliable negative samples generated by DDI-PULearn via comparing with randomly selected negative samples and all potential negative samples. We also demonstrate the superior prediction performance of DDI-PULearn by comparing with five state-of-theart methods. Finally, we apply DDI-PULearn to predict unobserved DDIs and verify the results in DrugBank.

### Components for PCA

**Fig. 1:**
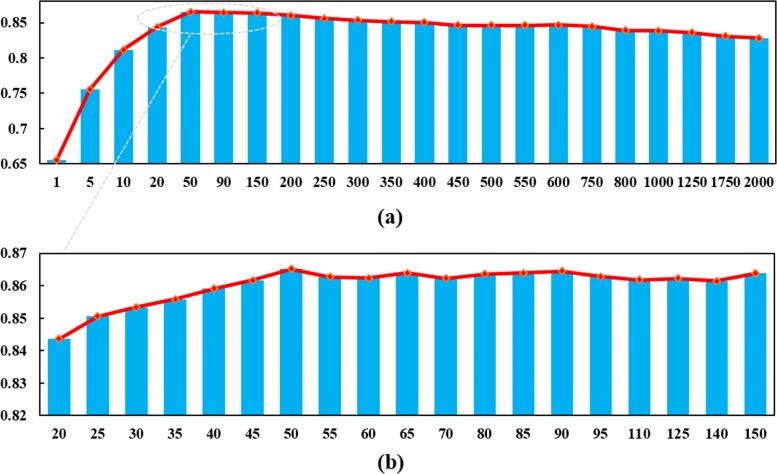
F1-scores of DDI-PULearn with different PCNs. The x-axis is the PCA component number and the y-axis is the F1-score. Panel (**a**) shows the F1-scores for PCN between 1 and 2000, and Panel (**b**) is an amplification of the range [20,150] (amplification ratio = 5)

To obtain the best setting for the PCA component number (PCN), we tried the following settings: *PCN*∈{1, 5, 10, 20, 30, 40, 50, 65, 80, 95, 110, 125, 140, 150, 160, 175, 200, 225, 250, 275, 300, 350, 400, 450, 500, 550, 600, 750, 800, 1000, 1250, 1750, 2000 }. The F1-scores of DDI-PULearn with different PCNs are illustrated in Fig. 1. It can be observed that the F1-score increases with *PCN* when *PCN*≤50. Besides, the F1-score values plateau when the PCN is larger than 50. The same conclusion can be drawn from the AUC results, as shown in Figure S1 in Additional file 1. Based on the above observation and considering the computational memory and time cost (computational memory and time increase with PCN), we set PCN as 50 for DDI-PULearn in our experiments.

### Representation of DDIs using multi-source drug property data

**Fig. 2:**
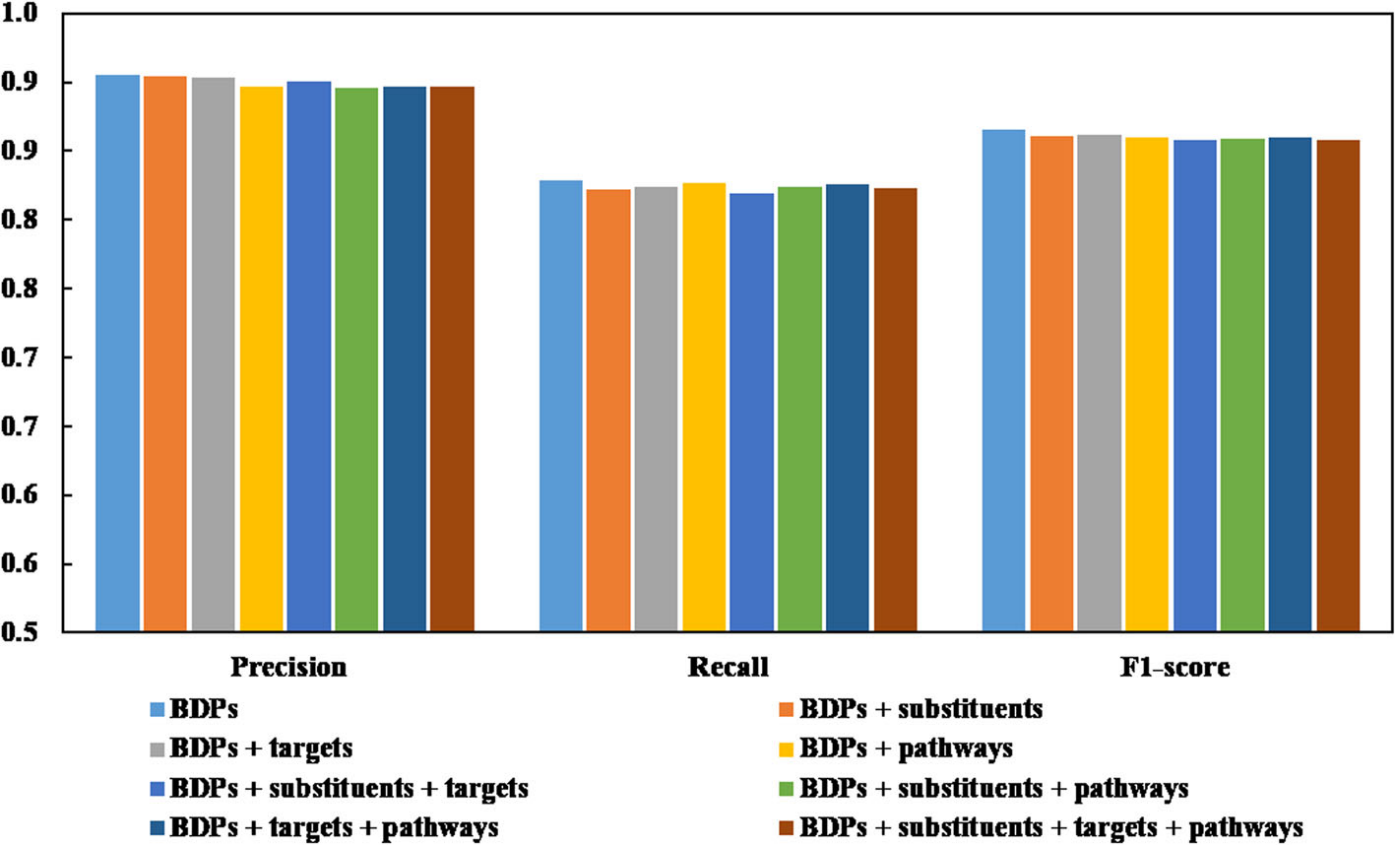
Prediction results using different combinations of drug features. BDPs refer to the basic drug properties namely drug chemical substructures, drug targets, and drug indications

As mentioned in the “Feature vector representation for DDIs” subsection, we perform the feature ranking analysis to decide which drug property to use for DDI representation. Here, we conduct more experiments to confirm the analysis results. Specifically, we use the drug chemical substructures, drug targets and drug indications as basic drug properties (BDPs) for representation. Then we test the following 8 combinations of drug features for predictions: (1) BDPs; (2) BDPs + substituents; (3) BDPs + targets; (4) BDPs + pathways; (5) BDPs + substituents + targets; (6) BDPs + substituents + pathways; (7) BDPs + targets + pathways; (8) BDPs + substituents + targets + pathways. Apart from the feature vector representation, other details of the eight combinations are the same with DDI-PULearn. Fig. 2 shows the bar charts of the prediction results. It can be observed that all performance evaluation indices (i.e., precision/recall/F1-score) vary very slightly among the above 8 combinations. Employing more drug features for predictions bring redundant information which doesn’t improve the prediction performance. It indicates that drug properties including drug substituents, drug targets and drug pathways play a minor role in the DDI predictions while the basic drug properties decide the prediction performance. The results further confirm the conclusion drawn in the previous feature ranking analysis. The detailed evaluation index values of the predictions are listed in Table S1 in Additional file 1.

### Performance improvement brought by identified reliable negative samples

Existing classification-based models either use all potential negative samples (all-negatives hereafter) or random negative samples (random-negatives hereafter) for predictions [5, 13]. All-negatives refer to all potential non-DDIs (i.e., unobserved DDIs) which are not in the positive samples. Random-negatives are generated by selecting a random number of negatives from all-negatives. To demonstrate the prediction performance improvement brought by reliable negative samples identified by DDI-PULearn, we compare DDI-PULearn with the above two baseline methods. Specifically, we obtain 101,294 ($C_{548}^{2}-48,584$) negatives for all-negatives. And we randomly select the same number of negatives (i.e., 45,026) with DDI-PULearn as random-negatives. Besides the negative samples, other details of prediction using all-negatives and random-negatives are the same with DDI-PULearn. To avoid bias, random-negatives are repeated 5 times and the average results are used for the final evaluation. Related prediction results are shown Table 1. It can be clearly seen that the prediction performances are significantly improved owing to the identified reliable negative samples. For example, the F1-score improvement over random-negatives and all-negatives are 0.147 (20.47%) and 0.315 (57.27%). It suggests that a better decision boundary has been learned with the identified reliable negative samples.

**Table 1:** Prediction performance comparison with the two baseline methods, namely all-negatives and random-negatives

Method	Precision	Recall	F1-score
DDI-PULearn	0.906	0.828	0.865
Random-negatives	0.765	0.676	0.718
All-negatives	0.709	0.449	0.550

### Comparison with existing state-of-the-art methods

To further confirm the superior performance of DDI-PULearn, we compare it with several state-of-the-art methods reported in a recent study [25] using the same dataset. Same as [25], we evaluated DDI-PULearn by 20 runs of 3-fold cross-validation and 5-fold cross-validation under the same condition. The macro-averaging results of the 20 runs are used for final evaluation. The comparison results are listed in Table 2. Vilar’s substructure-based method [6] and Vilar’s interaction-fingerprint-based method [4] are two similarity-based methods proposed by Vilar et al.; Zhang’s weighted average ensemble method, Zhang’s L1 classifier ensemble method and Zhang’s L2 classifier ensemble method are three ensemble methods which integrate neighbor recommendation, random walk and matrix perturbation by Zhang *et al* [25]. As shown in Table 2, DDI-PULearn achieves better performance than other state-of-the-art methods on all metrics. For example, using 5-fold cross-validation, DDI-PULearn outperforms the otherfive methods by 0.633 (276.6%), 0.415 (92.9%), 0.150 (21.1%), 0.139 (19.3%), 0.143 (19.9%) in F1-score respectively.

We also compared the proposed method with Hameed’s PU learning method [22]. Both our work research on the 548 benchmark drugs. We inferred 45,026 reliable negatives which cover all the 548 researched drugs. By contrast, Hameed inferred 589 negatives and just covers only 256 researched drugs. To fairly compare with Hameed’s method, we extracted the top 589 negatives in terms of inference scores from our inferred negatives and use the same strategy with Hameed to extract 589 random positives (hereinafter referred to as DDI-PULearn-Top).

**Table 2:** Performances of DDI-PULearn and the benchmark methods evaluated by 20 runs of 3-fold cross-validation and 5-fold cross-validation

Evaluation	Method	Precision	Recall	F1-score
3-fold CV	Vilar’s substructure-based method	0.145	0.535	0.229
	Vilar’s interaction-fingerprint-based method	0.377	0.553	0.447
	Zhang’s weighted average ensemble method	0.782	0.703	0.740
	Zhang’s L1 classifier ensemble method	0.788	0.717	0.751
	Zhang’s L2 classifier ensemble method	0.784	0.712	0.746
	DDI-PULearn	**0.902**	**0.822**	**0.860**
5-fold CV	Vilar’s substructure-based method	0.145	0.535	0.229
	Vilar’s interaction-fingerprint-based method	0.377	0.553	0.447
	Zhang’s weighted average ensemble method	0.775	0.659	0.712
	Zhang’s L1 classifier ensemble method	0.785	0.670	0.723
	Zhang’s L2 classifier ensemble method	0.783	0.665	0.719
	DDI-PULearn	**0.904**	**0.824**	**0.862**

We also constructed 10 training sets using the 589 top inferred negatives and randomly selected 589 known DDIs. The average performances of the 10 balanced training samples from 5-fold cross-validation are shown in Table 3. Note that SFR1 and SFR2 are two feature representation methods used by Hameed et al. [22]. It can be observed that DDI-PULearn-Top achieves comparable performance with Hameed’s GSOM-based PU learning methods. Specifically, DDI-PULearn-Top achieves better recall and F1-score than Hameed’s method using SFR1. It is slightly inferior to Hameed’s method using SFR2. Comparing with Hameed’s PU learning methods, DDI-PULearn has the following advantages: (1) DDI-PULearn infers many more negatives (45,026 vs 589) which is closer to the practical prediction task i.e., large-scale drug interaction prediction. Hameed’s inferred negatives cover part of researched drugs (256 from 589), thus only interactions between the covered drugs are predicted and evaluated. By contrast, our inferred negatives cover all researched drugs, the possible interaction between all researched drugs are predicted and evaluated. (2) The key goal of DDI-PULearn and Hameed’s method is to infer reliable negatives for classification. The 1178 evaluation samples (589 positives + 589 negatives) constructed by Hameed are quite few for the whole sample space ($C_{548}^{2}=149,878)$. Consequently, classifiers may not be able to learn enough knowledge to distinguish positive/negative from negative/positive for non-evaluation samples (148,700 = 149,878-1,178) though they perform well on the evaluation samples.

**Table 3:** Performance assessment of DDI-PULearn-Top and Hameed’s approaches using 10 training set and 5-fold cross-validation

Evaluation	DDI-PULearn-Top	Hameed’s GSOM-based PUL	
		SFR1	SFR2
Precision	0.944	0.951	0.974
Recall	0.934	0.861	0.975
F1-score	0.939	0.904	0.974

The above comparison results with existing state-of-the-art methods and another PU Learning method both demonstrate the superior performances and advantages of the proposed positive-unlabeled learning method DDI-PULearn.

### Novel DDIs predicted by DDI-PULearn

We employ DDI-PULearn to predict labels for the 101,294 unobserved DDIs, which are not available in the benchmark dataset. In the prediction, a larger prediction score of a drug pair suggests they have a higher interaction probability. We can obtain a recommendation list of novel DDIs by ranking them in descending order of their prediction scores. Like other data mining results, it is unrealistic to expect all highly ranked DDIs to be of value to domain experts. Therefore, we shortlist the top 25 novel interactions predicted by DDI-PULearn in Table 4. We further verify them in the DrugBank database which stores the latest DDI information. We highlight the confirmed DDIs in bold font. From Table 4, we can see that a significant ratio of predicted interactions is confirmed in DrugBank (11 out of 25). It indicates that DDI-PULearn does have the capability to predict novel drug-drug interactions.

**Table 4:** Top 25 novel DDIs predicted by the proposed method DDI-PULearn

Rank	Drug1	Drug1 Name	Drug2	Drug2 Name	Score
**1**	**DB01137**	**Levofloxacin**	**DB01182**	**Propafenone**	**1**
2	DB00512	Vancomycin	DB00704	Naltrexone	1
3	DB00704	Naltrexone	DB00783	Estradiol	1
4	DB00773	Etoposide	DB00783	Estradiol	1
5	DB01137	Levofloxacin	DB00704	Naltrexone	1
6	DB00635	Prednisone	DB00704	Naltrexone	1
**7**	**DB00295**	**Morphine**	**DB01037**	**Selegiline**	**0.975**
**8**	**DB01050**	**Ibuprofen**	**DB00712**	**Flurbiprofen**	**0.975**
**9**	**DB00218**	**Moxifloxacin**	**DB00328**	**Indomethacin**	**0.975**
10	DB00213	Pantoprazole	DB01189	Desflurane	0.975
11	DB00586	Diclofenac	DB01037	Selegiline	0.975
12	DB00398	Sorafenib	DB00445	Epirubicin	0.975
13	DB00724	Imiquimod	DB00331	Metformin	0.975
**14**	**DB00203**	**Sildenafil**	**DB00457**	**Prazosin**	**0.975**
**15**	**DB00999**	**Hydrochlorothiazide**	**DB00880**	**Chlorothiazide**	**0.975**
16	DB00635	Prednisone	DB00324	Fluorometholone	0.975
**17**	**DB00704**	**Naltrexone**	**DB00327**	**Hydromorphone**	**0.975**
**18**	**DB00295**	**Morphine**	**DB00704**	**Naltrexone**	**0.975**
**19**	**DB00813**	**Fentanyl**	**DB01232**	**Saquinavir**	**0.975**
**20**	**DB00624**	**Testosterone**	**DB00959**	**Methylprednisolone**	**0.95**
21	DB00959	Methylprednisolone	DB00550	Propylthiouracil	0.95
**22**	**DB01193**	**Acebutolol**	**DB00264**	**Metoprolol**	**0.95**
23	DB00295	Morphine	DB00674	Galantamine	0.95
24	DB01137	Levofloxacin	DB00323	Tolcapone	0.95
25	DB00591	Fluocinolone Acetonide	DB00641	Simvastatin	0.95

(DDIs which are confirmed in DrugBank are highlighted in bold font.)

## Discussions

Most existing methods are based on the closed-world assumption, taking validated interacted drug pairs as positives and unlabeled drug pairs as negatives to perform the prediction directly [4*–*7*,*^13^]. However, drugs from the unlabeled drug pairs still have considerable probabilities to interact. It means that the assumed negatives may include a considerable number of real positives which are yet unknown. As a result, classifiers trained with unlabeled drug pairs as negatives cannot learn a good boundary to classify true positives and true negatives.

Instead of taking unlabeled drug pairs as negatives directly, we develop a PU-Learning method to generate reliable negatives by learning from the positive and unlabeled samples. The comparison experiments with two baseline methods, five state-of-the-art methods, and a PU-learning method demonstrate that DDI-PULearn achieves superior performance. Investigation on the top-predicted novel DDIs also shows the competence of DDI-PULearn on predicting novel DDIs. The superior performance of DDI-PULearn can be attributed to the following aspects: (1) In the first step of generating reliable negative seeds, it takes advantage of the converse negative proposition of the similarity-based methods (achieved remarkable performance), i.e., dissimilar drugs are less likely to interact. It also utilizes the advanced one-class learning technique OCSVM. The combination of the above two techniques ensures that the most reliable negative seeds are generated. (2) In the second step, SVM trained with validated positives and the generated negative seeds is employed to predict the remaining unlabeled drug pairs. Then, the newly predicted negatives are added to the negative set to train SVM for the next round prediction. The process is repeated iteratively until no new negatives are obtained. The initial training with reliable negative seeds ensures the classification boundary is properly learnt and the iterative process extracts all possible negatives. Through the above learning from the validated positive samples and unlabeled samples, a better classification boundary has been learnt.

## Conclusions

In this work, we propose a novel positive-unlabeled learning method named DDI-PULearn for large-scale drug-drug interaction predictions. DDI-PULearn first generates seeds of reliable negative samples from the unlabeled samples using two techniques namely OCSVM and KNN. Then trained with the generated seeds, DDI-PULearn employs SVM to identify all reliable negative samples iteratively. Following that, DDI-PULearn represents the labeled positive samples and identified negative samples as vectors by a similarity-based representation method using abundant drug properties. Finally, the vectors are compressed via PCA and further used as input for binary classifications. The innovation of this work lies in the design of the novel PU-Learning method and in the method for DDI representations. In the experimental part, we discussed the determination of PCA components number and different drug properties for DDI representations. We demonstrate the superior performance of DDI-PULearn by comparing it with two baseline methods and five state-of-the-art methods. All experimental results show that the DDI prediction performance is significantly improved owing to DDI-PULearn. Besides, results for prediction of novel DDIs suggest that DDI-PULearn is competent to identify novel DDIs.

DDI-PULearn is useful in various areas and able to guide drug development at different stages. For instance, at the early stage of drug candidate selection, DDI-PULearn can help to decide whether the drug molecules should be dropped or kept for further study. In addition, warnings about the potential interactions which may cause serious side-effects can be given to the public on time.

## Methods

### Data resources

#### Drug properties

We extract drug properties from different data sources. Drug chemical substructures and drug substituents are extracted from DrugBank [26], a comprehensive drug database. Drug targets are obtained by fusing drug-target associations from both DrugBank and DrugCentral [27]. The drug-side-effect associations are downloaded from SIDER [28], a large labeled side-effect database. The drug-indication associations, drug-pathway associations, and drug-gene associations are retrieved from the CTD (comparative toxicogenomics database) [29].


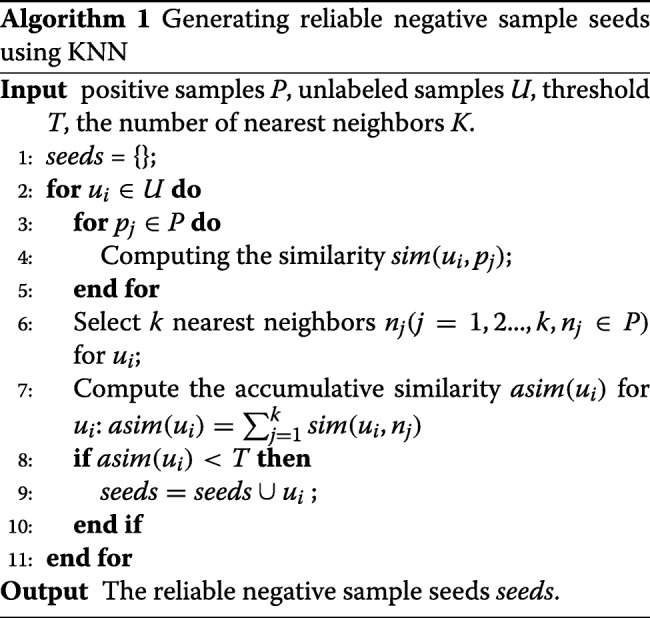


#### Drug-drug interactions

We use a recent benchmark dataset [25] collected from TWOSIDES [30], a database which contains DDIs mined from FAERS. It contains 548 drugs and 48,584 pairwise drug-drug interactions. The specific drug list and all verified DDIs are available in Additional file 2.

### Proposed methods

**Fig. 3:**
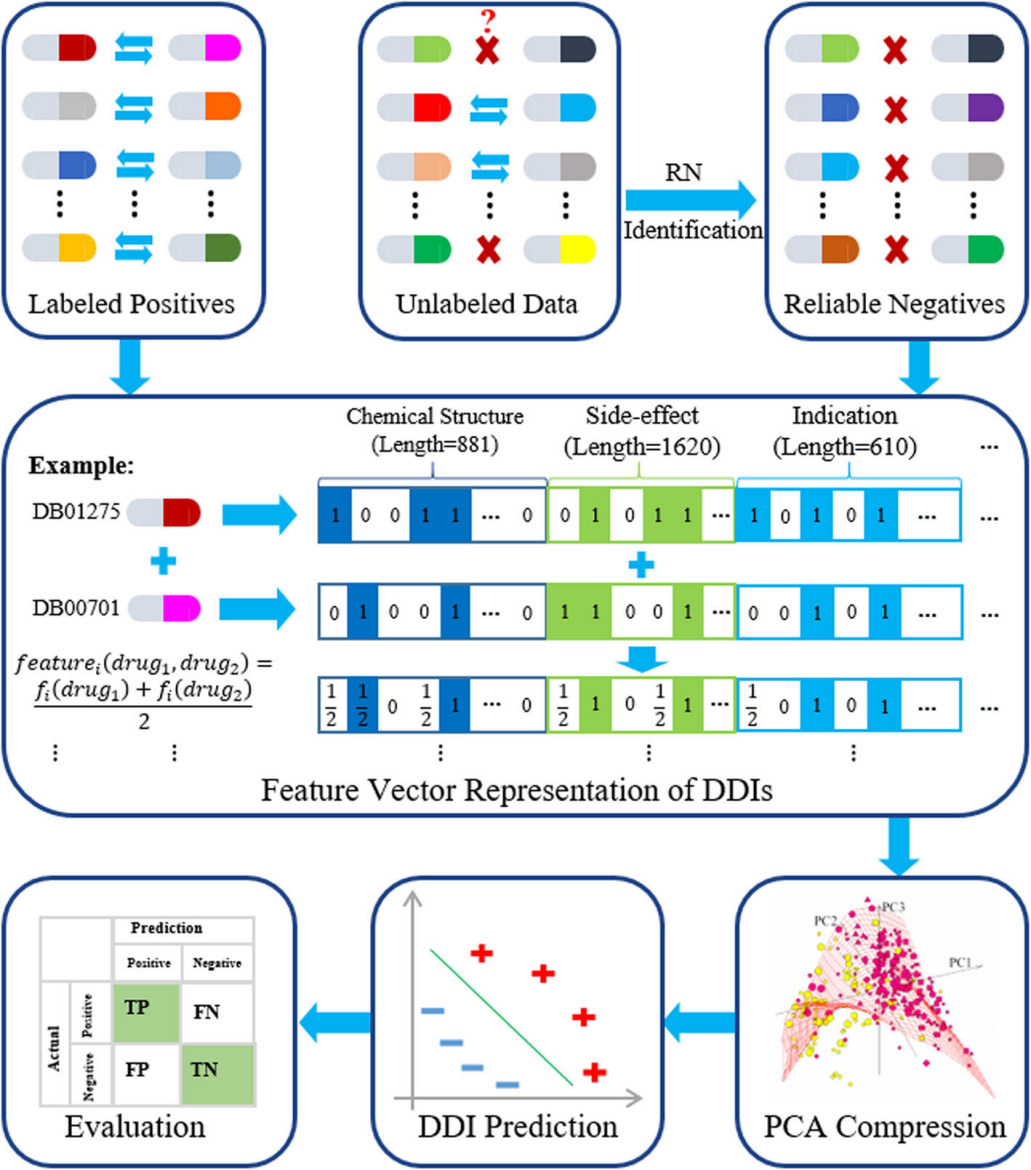
The framework of the proposed method. It consists of the following five components: reliable negative sample identification, feature vector representation for DDIs, PCA compression, DDI prediction, and performance evaluation. RN: reliable negative samples; PCA: principal component analysis; DDI: drug-drug interaction

The framework of the proposed method is illustrated in Fig. 3. It consists of five components listed as follows: reliable negative sample identification, feature vector representation for DDIs, PCA compression, DDI prediction, and performance evaluation. First, reliable negative samples are generated using DDI-PULearn. Then both the labeled positive samples and the reliable negative samples are represented as vectors according to the drug properties, such as chemical substructures, associated side-effects, and indications. Next, the sample vectors are compressed into a lower-dimension space using PCA. Following that, the compressed vectors together with their labels are used as input for DDI prediction. Finally, the prediction performance is evaluated according to the confusion matrix.

#### Reliable negative sample identification

**Fig. 4:**
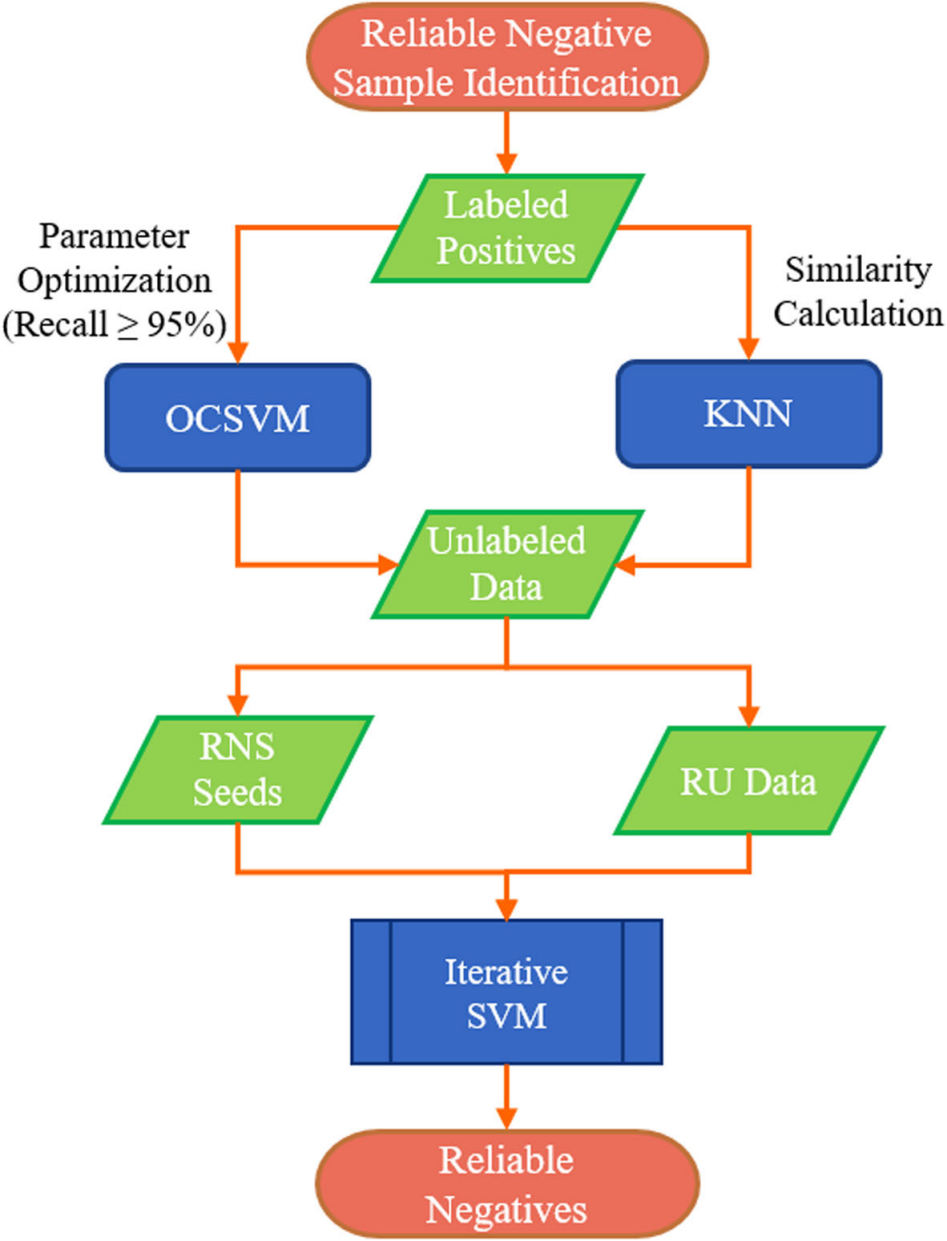
The flow chart for the identification of reliable negative samples. OCSVM: one-class support vector machine; KNN: k-nearest neighbor; RNS: reliable negative samples; RU: remaining unlabeled

We propose a novel two-step strategy to generate reliable negative samples. In the first step, we generate RNS seeds from the unlabeled samples using OCSVM and KNN. Then we employ SVM trained with labeled positive samples and RNS seeds to generate reliable negative samples iteratively. Labeled positive samples are validated DDIs and unlabeled samples are unobserved DDIs between every two drugs which are not in labeled positive samples. Fig. 4 details the flow for identification of reliable negative samples.

A. RNS seed generation

In the first step, we employ two techniques namely OCSVM and KNN to generate the RNS seeds. For OCSVM, we feed it with all labeled positive samples and optimize its parameters via 5-fold cross-validation. To ensure that the majority of true DDIs are correctly predicted, a high recall (>0.95) is required for OCSVM. With the optimized parameter settings (nu: 0.05, gamma: 0.001), OCSVM achieves a recall of 0.951 and generates 1,602 RNS seeds from the 101,294 ($C_{548}^{2}$-48,584) unlabeled samples.

As described in the next subsection, each DDI is represented as a 3,111-dimensional vector. We use the cosine function as the similarity measure for KNN:

1 $$ {\begin{aligned} sim({ddi}_{i}, {ddi}_{j}) &= cosine(vector({ddi}_{i}), vector({ddi}_{j}))\\&=\frac{\sum_{l=1}^{3,111}{[{vector}_{l}({ddi}_{i})*{vector}_{l}({ddi}_{j})]}}{\sum_{l=1}^{3,111}{vector_{l}({ddi}_{i})^{2}}*\sum_{l=1}^{3,111}{vector_{l}({ddi}_{j})^{2}}} \end{aligned}}  $$

where *vector*(*ddi*_*i*_) and *vector*(*ddi*_*j*_) are vectors of the DDI/sample *ddi*_*i*_ and *ddi*_*j*_ respectively. The specific process to generate RNS seeds using KNN is described in Algorithm 1. After optimizing, we set *k* as 5 and the threshold as 4.026. Using the KNN strategy, we obtain 5000 RNS seeds. Merging the RNS seeds generated by OCSVM and KNN, we finally obtain 6602 RNS seeds (see Table S6 in Additional file 2).

B. Iterative SVM for RNS identification

In the second step, we run SVM trained by labeled positive samples and RNS seeds iteratively to identify all reliable negatives from the remaining unlabeled data. The pseudo-code is shown in Algorithm 2. We aim to identify all reliable negative samples from the unlabeled data, thus we use the last SVM classifier at convergence as the best classifier instead of selecting a good classifier from the classifiers built by SVM. Through the iteration, we finally obtained 45,026 reliable negative samples.


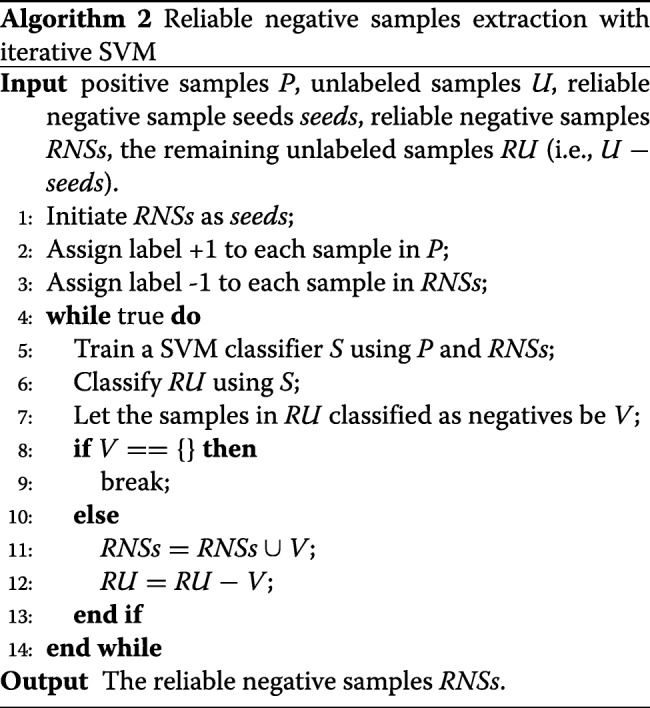


#### Feature vector representation for DDIs

We collected a variety of drug properties which may help to improve the prediction, namely drug chemical substructures, drug substituents, drug targets, drug side-effects, drug indications, drug-associated pathways, and drug-associated genes. We investigate which drug property to use for drug representation by feature importance ranking using Random Forrest. The implementation details and experiment results are described in Additional file 1. The feature ranking analysis shows that drug properties including drug chemical substructures, drug targets, and drug indications play a leading role in DDI prediction, thus, we decide to employ them for drug representation. Specifically, we represent each drug as a 3111-dimensional feature vector using 881 drug chemical substructures, 1620 side-effects, and 610 indications. The drug chemical substructures correspond to 881 substructures defined in the PubChem database [31]. The side-effects and indications are 1,620 unique side-effects in SIDER [28], and 610 unique indications in DrugBank [26] respectively. Each bit of the feature vector denotes the absence/presence of the corresponding substructure/side-effect/indication by 0/1. Further, we propose a similarity-based representation for DDIs based on the following formula:

2 $$ \begin{aligned} {vector}_{k}({drug}_{i}, {drug}_{j}) = \\\frac{feature_{k}({drug}_{i}) + {feature}_{k}({drug}_{j})}{2} \end{aligned}  $$

where *feature*_*k*_(*drug*_*i*_) and *feature*_*k*_(*drug*_*j*_) are the *k*-th bit of the feature vectors of drug *drug*_*i*_ and *drug*_*j*_ respectively, *vector*_*k*_ is the *k*-th bit of vector for the DDI *drug*_*i*_- *drug*_*j*_.

#### PCA compression

There are 149,878 $\left (C_{548}^{2}\right)$ possible DDIs between the 548 drugs used for experiments. Thus the size of the classification input could be around the order of magnitude of billion (149,878∗3,111). Such high dimensionality inevitably incurs a huge computational cost. To speed up the prediction process, we employ PCA to map the raw vectors of DDIs into lower-dimension space. Specifically, all training DDI vectors are used to fit the PCA first. Then the fitted PCA is used to transform both the training and testing DDI vectors into lower-dimensional vectors. Finally, the compressed vectors are used as input to train and validate the binary classifier.

#### DDI prediction

We formalize the DDI prediction task as a binary classification problem to predict a DDI is true or not. The inputs for the binary classifiers are the compressed vectors of DDIs and their labels. Specifically, we label labeled positive samples (i.e., validated DDIs) as +1 and the generated reliable negative samples as -1. Finally, we train and test a binary classifier with the above vectors and labels. We employ “Random Forrest" as the binary classifier in this work.

#### Performance evaluation

5-fold CV (cross-validation) is performed to evaluate the prediction performance: (i) DDIs in the gold standard set are split into 5 equal-sized subsets; (ii) each subset is used as the test set, and the remaining 4 subsets are taken as the training set in turn to train the predictive models; (iii) the final performance is evaluated on all results over 5-folds. To avoid the bias of data split, 5 independent runs of 5-fold CV are implemented and average results are used for final evaluation. Precision, recall, F1-score, and AUC (area under the receiver operating characteristic curve) are used as evaluation metrics.

## Supplementary information

**Additional file 1** The supplementary results for this work.
• “Feature importance ranking using Random Forrest" : implementation details and experiment results of the feature importance ranking analysis using Random Forrest.
• Figure S1: AUCs of DDI-PULearn with different PCNs (PDF 316 kb).

**Additional file 2** This file contains lists of researched drugs, verified DDIs, reliable negative samples generated by DDI-PULearn, and the detailed feature importance ranking results.
• Table S1: DDI prediction results using different combinations of drug features.
• Table S2: 548 drugs researched in this work.
• Table S3: 45,026 reliable negative samples generated by DDI-PULearn.
• Table S4: 48,584 verified DDIs in the benchmark dataset.
• Table S5: Detailed feature importance ranking results by Random Forrest.
• Table S6: 6602 reliable negative sample seeds generated by OCSVM and KNN (XLSX 1,661 kb).

## Data Availability

The data used in this study all are available in the Additional files.

## Abbreviations

AUC: Area under the receiver operating characteristic curve
BDPs: basic drug properties
CTD: Comparative toxicogenomics database
CV: Cross validation
DDI-PULearn: The proposed PU learning method
EM: Expectation maximization
FAERS: Food and drug administration adverse event reporting system
KNN: k-nearest neighbors
NB: Naive Bayesian
OCSVM: One-class support vector machine
PCN: PCA component number
PU learning: Positive and unlabeled learning
RNSs: Reliable negative samples
SOM: Self organizing map
SVM: Support vector machine
