# Supplementary material for: DDI-PULearn: a positive-unlabeled learning method for large-scale prediction of drug-drug interactions
Source: BMC Bioinformatics. 2019 Dec 24;20(Suppl 19):661. doi: 10.1186/s12859-019-3214-6 (PMC6929327; doi:10.1186/s12859-019-3214-6)
Supplement: Supplementary file 1 — Additional file 1 The supplementary results for this work. • “Feature importance ranking using Random Forrest" : implementation details and experiment results of the feature importance ranking analysis using Random Forrest. • Figure S1: AUCs of DDI-PULearn with different PCNs (PDF 316 kb). [file 12859_2019_3214_MOESM1_ESM.pdf]

DDI-PUlearn: a novel positive-unlabeled learning method for large-scale prediction of drug-drug interactions

Yi Zheng[1], Hui Peng[1], Xiaocai Zhang[1], Zhixun Zhao[1], Xiaoying Gao[2], and Jinyan Li[1, *]

[1] Advanced Analytics Institute, University of Technology Sydney, PO Box 123, Broadway, NSW 2007, Australia.

[2] School of Engineering and Computer Science, Victoria University of Wellington, Cotton Building, Kelburn Campus, Wellington, 6140, New Zealand.

*** Corresponding Author E-mail: Jinyan.Li@uts.edu.au**

1. Additional information introduction

There are 2 additional files in total. The files are structured as follows:

(1) Additional file 1

A. The implementation details and experiment results of the feature importance ranking analysis.

B. Figure S1: AUCs of DDI-PULearn with different PCNs.

(2) Additional file 2

Additional results and data used in this work.

A. Table S1: DDI prediction results using different combinations of drug features.

B. Table S2: 548 drugs researched in this work.

C. Table S3: 45,026 reliable negative samples generated by DDI-PULearn.

D. Table S4: 48,584 verified DDIs in the benchmark dataset.

E. Table S5: Detailed feature importance ranking results by Random Forrest.

F. Table S6: 6,602 reliable negative sample seeds generated by OCSVM and KNN.

2. Feature importance ranking using Random Forrest

In this work, we collected a variety of drug property data that may help to improve the prediction performance, i.e., drug chemical substructure, drug substituents, drug target proteins, drug side-effects, drug indications, drug-associated pathways, and drug-associated genes. Then we investigate how these drug data contribute to the performance of drug-drug interactions using Random Forrest (RF). Random

Forest is a widely-used method for feature ranking because it requires very little feature engineering and parameter tuning [1].

Similar with the subsection "Feature vector representation for DDIs", we first represent each drug as a 15,753-dimensional feature vector according to its property data, including 881 drug chemical substructures, 1,235 drug substituents, 722 drug targets, 1,685 drug associated pathways, 9,000 drug associated genes, 1,620 drug side-effects and 610 drug indications. The drug chemical substructures correspond to 881 substructures defined in the PubChem database; The drug substituents, drug targets, and drug indications are 1,235 unique substituents, 722 unique targets and 610 unique indications in DrugBank respectively; The drug associated pathways and genes are 1,685 unique pathways and 9,000 unique genes in CTD; The side-effects are 1,620 unique side-effects in SIDER. Each bit in the feature vector denotes the absence/presence of the corresponding substructure/substituent/target/side-effect/indication/pathway/gene by 0/1. Then each drug is represented as a 15,753-dimension feature vector by concatenating the above features sequentially. Following that, we represent each drug-drug interaction as the average value of the two corresponding drug feature vectors using formula 2. Finally, we treat all validated DDIs as positive samples (48,584), and all potential DDIs which are not validated DDIs as negative samples ($10{,}1294 = C_{548}^{2} - 48{,}584$). The RF is employed as the classifier to classify the above samples and the feature importance scores are produced by RF.

The detailed results are included in Table S5 in Additional file 2. Here, we provide the statistical information for the feature importance ranking as shown in Table 1 and Table 2. We rank all the 15,753 features in descending order of their RF feature importance scores. We observed the feature numbers belong to different drug properties among the top 50/100/150/200/300/400/500/600/700/800/900/1000 ranked features. It can be seen from Table 1 that features from drug properties including chemical substructures, indications, and side-effects account for most of the top-ranked features. For example, for the top 50 features, they take 47 positions (94%); for the top 1000 features, they take 707 positions (70.7%). The feature number belongs to different drug properties varies a lot. Drug properties which have more features are more likely to take more positions in the top-ranked features. To avoid this bias, we also investigate the ratio of top-ranked features relative to the total feature number for each drug property. For each drug property, the ratio is the quotient of the top-ranked features belong to this drug property and the total number of features belong to this property. Related results are listed in table 2. It can be seen that features belong to the above three properties (i.e., chemical substructures, indications, and side-effects) achieve larger ratios as well. All the above results indicate that features from the chemical substructures, indications, and side-effects play a leading role in the DDI prediction task. Therefore, we use chemical substructures, indications, and side-effects as base properties to represent drugs for DDI-PUlearn. More experiments for investigation on the impacts of drug properties in the DDI prediction performance are described in the main text.

Table 1. The feature importance ranking for 7 drug features.

	**50**	**100**	**150**	**200**	**300**	**400**	**500**	**600**	**700**	**800**	**900**	**1000**
**chemical structure**	11	21	29	39	63	85	112	140	160	183	201	224
**indication**	3	5	13	15	25	35	42	50	56	63	74	82
**side effect**	33	64	85	114	160	198	233	265	304	335	370	401
**substituent**	1	3	4	4	11	20	25	36	44	55	66	72

**target**	0	0	2	2	2	2	2	5	6	8	11	16
**pathway**	2	5	11	16	26	40	54	64	85	100	116	133
**gene**	0	2	6	10	13	20	32	40	45	56	62	72

Table 2. The feature importance ranking percentage for 7 drug features.

	**50**	**100**	**150**	**200**	**300**	**400**	**500**	**600**	**700**	**800**	**900**	**1000**
**chemical structure**	1.2%	2.4%	3.3%	4.4%	7.2%	9.6%	12.7%	15.9%	18.2%	20.8%	22.8%	25.4%
**side effect**	0.5%	0.8%	2.1%	2.5%	4.1%	5.7%	6.9%	8.2%	9.2%	10.3%	12.1%	13.4%
**indication**	2.0%	4.0%	5.2%	7.0%	9.9%	12.2%	14.4%	16.4%	18.8%	20.7%	22.8%	24.8%
**substituent**	0.1%	0.2%	0.3%	0.3%	0.9%	1.6%	2.0%	2.9%	3.6%	4.4%	5.3%	5.8%
**target**	0.0%	0.0%	0.3%	0.3%	0.3%	0.3%	0.3%	0.7%	0.8%	1.1%	1.5%	2.2%
**pathway**	0.1%	0.3%	0.7%	0.9%	1.5%	2.4%	3.2%	3.8%	5.0%	5.9%	6.9%	7.9%
**gene**	0.0%	0.0%	0.1%	0.1%	0.1%	0.2%	0.4%	0.4%	0.5%	0.6%	0.7%	0.8%

3. Figure S1

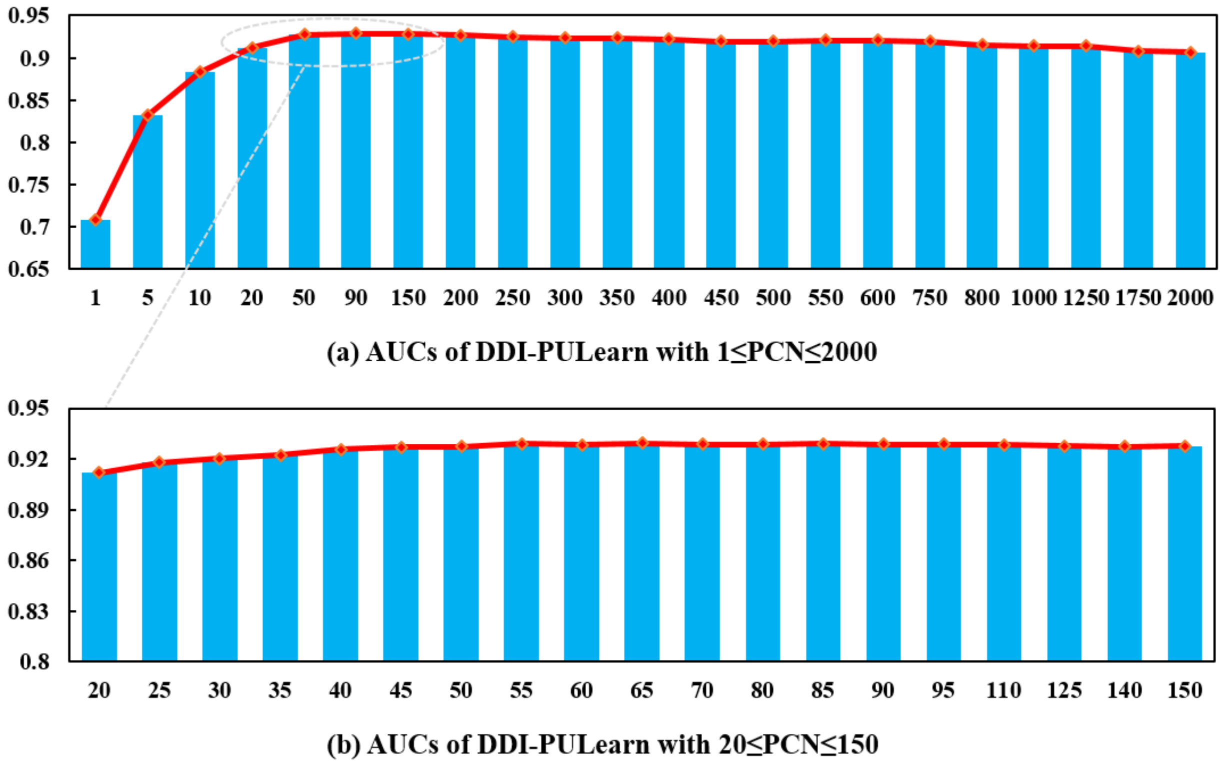

Figure S1. AUCs of DDI-PULearn with different PCNs. The x-axis is the PCA component number and the y-axis is the F1-score. Panel (a) shows the AUCs for PCN between 1 and 2,000, and Panel (b) is an amplification of the range [20,150].

Reference

[1] Menze, B. H., Kelm, B. M., Masuch, R., Himmelreich, U., Bachert, P., Petrich, W., & Hamprecht, F. A.: A comparison of random forest and its Gini importance with standard chemometric methods for the feature selection and classification of spectral data. BMC bioinformatics, 10(1), 213 (2009).
